# Supplementary material for: Degradation of a New Herbicide Florpyrauxifen-Benzyl in Water: Kinetics, Various Influencing Factors and Its Reaction Mechanisms
Source: Int J Mol Sci. 2023 Jun 23;24(13):10521. doi: 10.3390/ijms241310521 (PMC10342155; doi:10.3390/ijms241310521)
Supplement: Supplementary file 1 [file ijms-24-10521-s001.zip › ijms-2363117-supplementary.pdf]

International Journal of *Molecular Sciences*

MDPI

## Supplementary Material

# Degradation of a New Herbicide Florpyrauxifen-benzyl in Water: Kinetics, Various Influencing Factors and Its Reaction Mechanisms

**Rendan Zhou [1], Zemin Dong [1,2], Long Wang [1], Wenwen Zhou [3], Weina Zhao [2], Tianqi Wu [1], Hailong Chang [1], Wei Lin [1] and Baotong Li [1,*]**

**Table S1.** Temperature effect coefficient (*Q*), activation energy (*E*a), activation enthalpy (Δ*H*), activation entropy (Δ*S*) and Gibbs free energy (Δ*G*) of florpyrauxifen-benzyl hydrolysis.

| Temperature/K | Rate Constant (*k*)/d$^{-1}$ | *Q* | *E*a / kJ mol$^{-1}$ | Δ*H* / kJ mol$^{-1}$ | Δ*S* / kJ (mol K)$^{-1}$ | Δ*G* / kJ mol$^{-1}$ |
|---|---|---|---|---|---|---|
| 288 | 0.0086 | / | 140.0197 | 137.6253 | 0.2960 | 52.3798 |
| 298 | 0.0529 | 6.1739 | 140.0197 | 137.5421 | 0.2957 | 49.4213 |
| 308 | 0.3573 | 6.7530 | 140.0197 | 137.4590 | 0.2954 | 46.4656 |
| 323 | 4.6981 | / | 140.0197 | 137.3343 | 0.2950 | 42.0371 |

**Table S2.** Information on the disposable face masks (DFMs).

| Brand | Mass/g | Retail Price/CNY [a] | Production Date | Executive Standard | Materials |
|---|---|---|---|---|---|
| JIEMIAN | 2.8126~2.9325 | 0.3 | 2022.03 | YY0469-2011 | PP/PET |
| Size / cm | Color | | | | |
| 17.5× 9.5-three layers | Blue/White | | | | |

Note: a: We divided the total purchase price by the number of the masks.

**Table S3.** Composition of artificial seawater.

| Metal Salts | Amount/mg L$^{-1}$ |
|---|---|
| Sodium chloride, NaCl | 24.3200 |
| Magnesium chloride, $MgCl_2$ | 5.1400 |
| Calcium chloride anhydrous, $CaCl_2$ | 1.1400 |
| Potassium chloride, KCl | 0.6900 |
| Sodium bicarbonate, $NaHCO_3$ | 0.2000 |
| Potassium bromide, KBr | 0.1000 |
| Boric acid, $H_3BO_3$ | 0.0270 |
| Strontium chloride hexahydrate, $SrCl_2 \times 6H_2O$ | 0.0260 |
| Ammonium chloride, $NH_4Cl$ | 0.0064 |
| Sodium fluoride, NaF | 0.0030 |
| Sodium metasilicate, $Na_2SiO_3$ | 0.0020 |
| Iron(III) phosphate tetrahydrate, $FePO_4 \times 4H_2O$ | 0.0010 |

The artificial seawater solution pH is 8.18.

**Table S4.** The mass spectrometry conditions for the chromatographic analysis of florpyrauxifen-benzyl.

| Compound | Retention Time/min | Selected Ion | Precursor Ion/m/z | Extracted Fragment Ion/m/z | DP/V | CE/eV | Ionization Mode |
|---|---|---|---|---|---|---|---|
| florpyrauxifen-benzyl | 5.62 | $[M+H]^+$ | 439.0422 | 50.0157 | 90 | 145 | + |
| | | | | 51.0235 | 90 | 145 | |
| | | | | 63.0236 | 90 | 140 | |
| | | | | 65.0387 | 90 | 95 | |
| | | | | 91.0551* | 90 | 95 | |
| **Ion source temperature** | **Spray voltage** | **Capillary temperature** | Gas1 | Gas2 | **Curtain Gas** | **EPI scan speed** | **Acquisition time** |
| /°C | /V | /°C | / psi | /psi | /psi | /Da/s | /ms |
| 550 | 5500 | 320 | 50 | 55 | 35 | 10000 | 100 |

where "*" represented quantitative ion, DP represented declustering potential and CE represented collision energy.

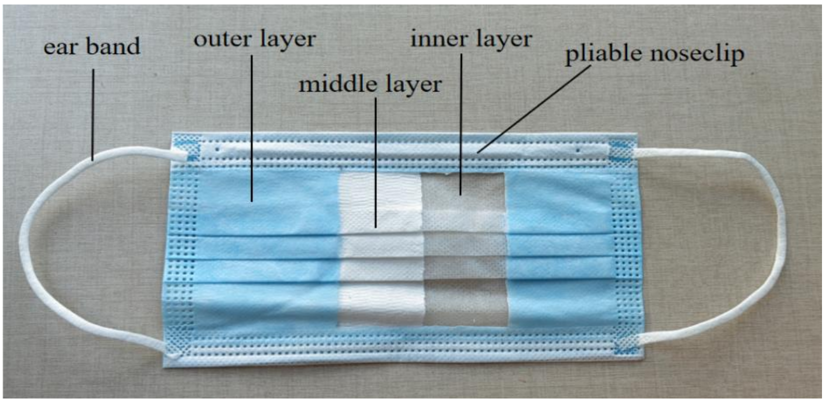


**Figure S1.** The components of disposable face masks (DFMs).

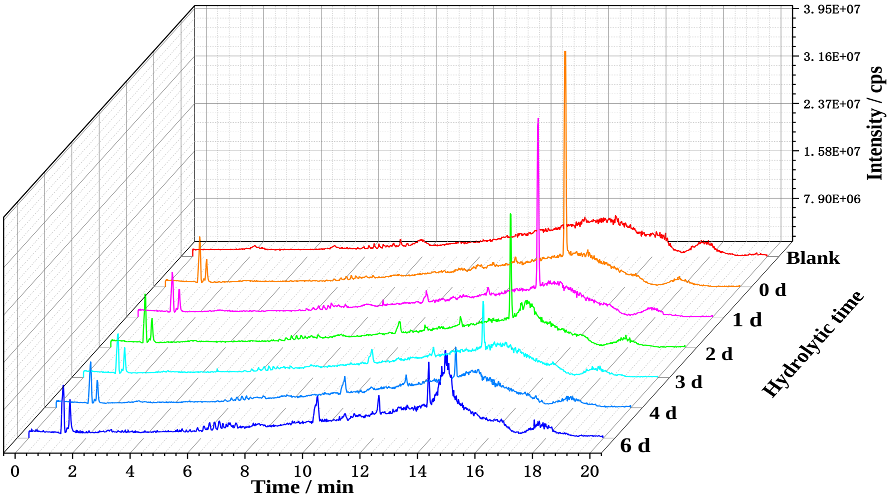


**Figure S2.** The total ion chromatograms of florpyrauxyfen-benzyl degradation solution performed. by QTOF-MS/MS.

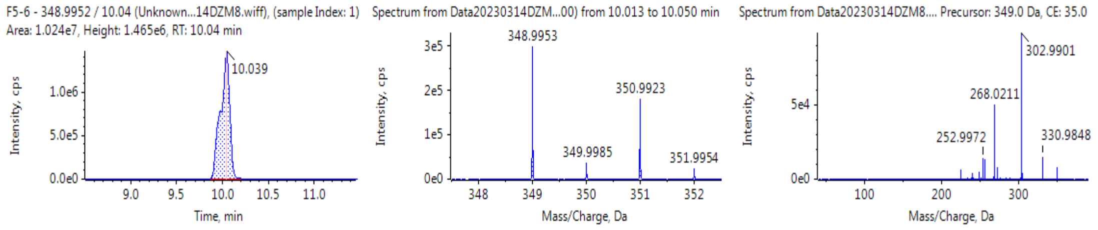


Product a:

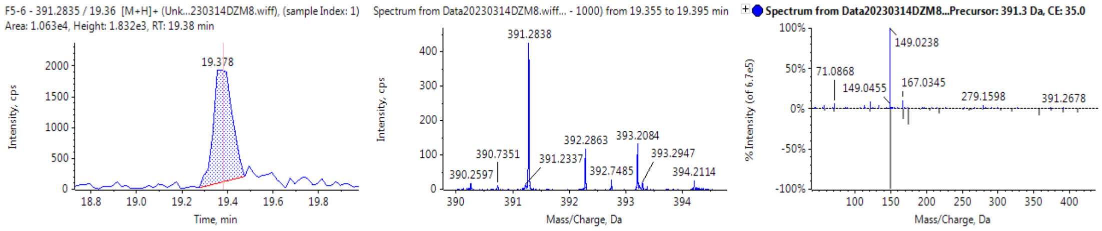


Product b:

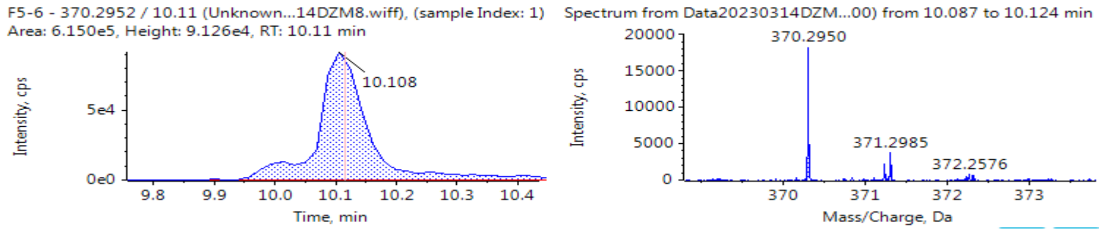


Product c:

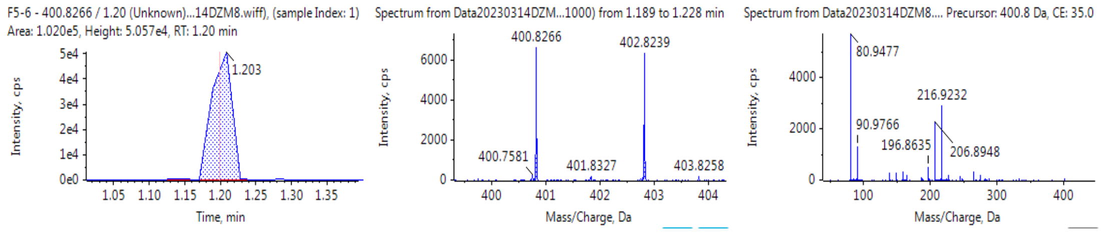


Product d:

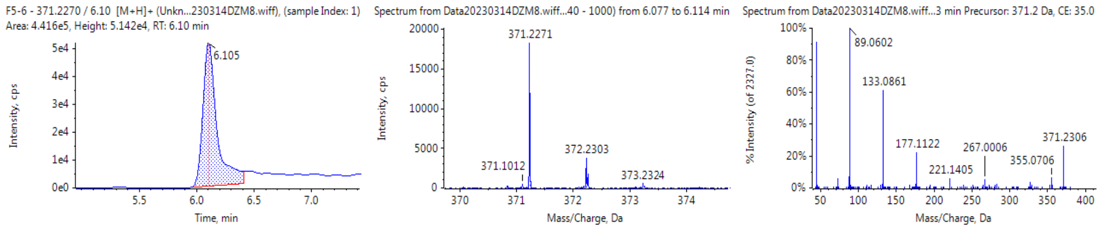


Product e:

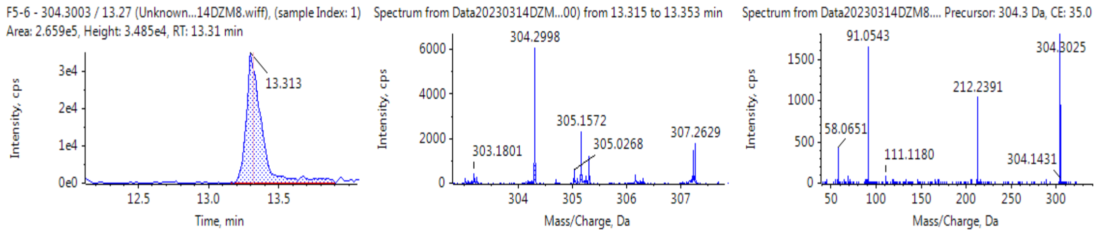


Product g:

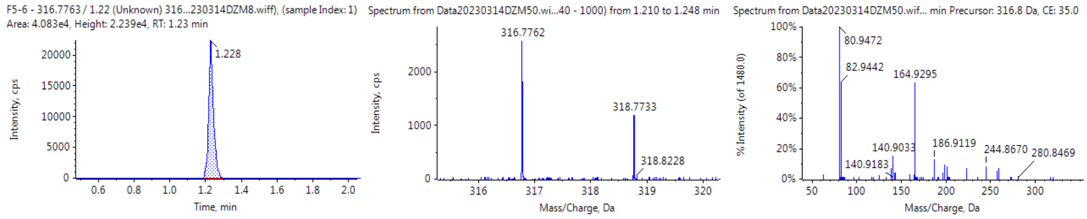


Product h:

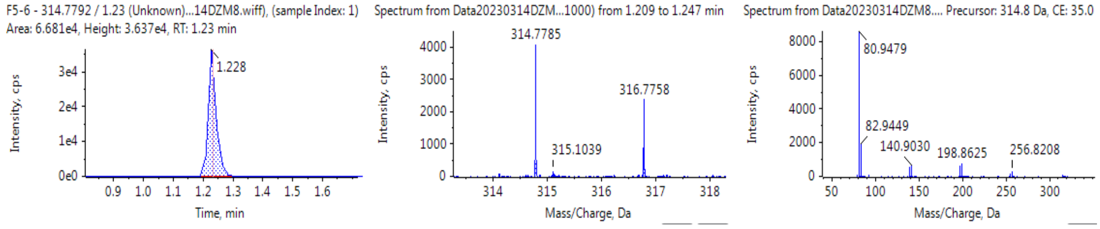


Product i:

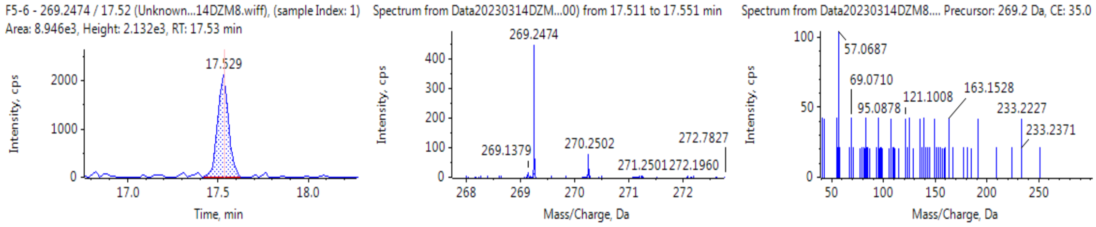


Product j:

**Figure S3.** The primary and secondary mass spectrograms of the hydrolytic products.

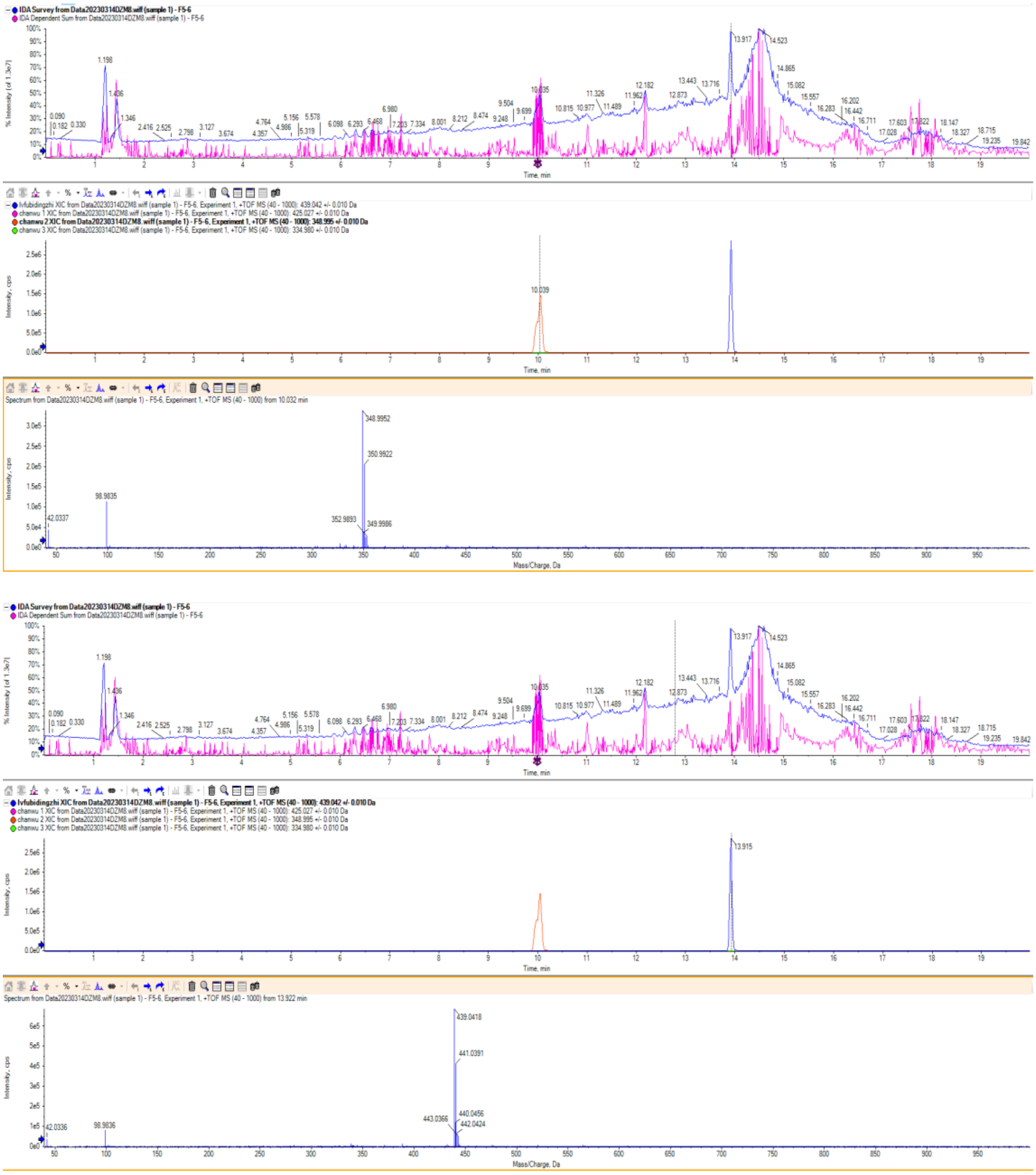


**Figure S4.** The total ion chromatograms and extraction ion chromatograms of florpyrauxifen-benzyl hydroxy and florpyrauxifen-hydroxy acid, primary mass spectrograms of florpyrauxifen (10.039 min) and florpyrauxifen-benzyl (13.915 min).
